# Strengthening sense of coherence: Evidence from a physical activity intervention targeting vulnerable adults

**DOI:** 10.1016/j.pmedr.2021.101554

**Published:** 2021-09-08

**Authors:** Kristina Thompson, Marion Herens, Johan van Ophem, Annemarie Wagemakers

**Affiliations:** a Department of Health Sciences, Vrije Universiteit Amsterdam, Amsterdam, the Netherlands; b Wageningen Centre for Development Innovation (WCDI), Wageningen University & Research, Wageningen, the Netherlands; c Urban Economics Group, Wageningen University & Research, Wageningen, the Netherlands; d Health and Society Group, Wageningen University & Research, Wageningen, the Netherlands

**Keywords:** Sense of coherence, Physical activity intervention, Community-based, Health promotion, Experiential learning, Salutogenesis, Socially vulnerable groups

## Abstract

Sense of coherence (SOC), a concept that refers to individuals’ abilities to manage, comprehend, and find meaning in their lives and the world around them, has been shown to be an important predictor of health outcomes. While SOC was initially hypothesized to be static after early-adulthood, there is growing evidence that health interventions can strengthen SOC. In this study, we accordingly examined whether SOC could be strengthened among adults in the context of a physical activity intervention.

This intervention, Communities on the Move, was conducted in the Netherlands, and was primarily targeted at older adults from socially vulnerable backgrounds. Four cohorts were followed for 18 months each, between 2012 and 2016. The SOC-3 questionnaire was used to collect data on SOC at baseline (T_0_) and after eighteen months (T_3_), with information on 117 participants in both of these waves. To assess the change in SOC between T_0_ and T_3_, ordered logistic regressions were performed, as well as mixed ordered logistic regressions with random intercepts for group and program location.

This study found evidence that SOC significantly changed from T_0_ to T_3_. Participants with weak SOC at baseline reported a median one-point stronger SOC at T_3_ (on a 6-point scale), while those with moderate or strong SOC at baseline reported a median change of zero points between T_0_ and T_3_. Further, based on the results of the regression analyses, those with weaker SOC scores were most likely to have stronger SOC at T_3_: having a weak SOC at baseline was associated with a 76% probability of stronger SOC, and a 4% probability of weaker SOC at T_3_. These results indicated that SOC may be strengthened in vulnerable older adults, particularly when their SOC is initially low.

## Highlights

• Sense of coherence (SOC) was stronger after a physical activity intervention.
• Adults’ SOC may be strengthened, particularly when SOC is initially low.
• SOC may be an alternative success indicator for physical activity interventions.

## 1 Introduction

Sense of coherence (SOC) is a concept that refers to individuals’ abilities to manage, comprehend, and find meaning in their lives and the world around them (Antonovsky, 1987, Super et al., 2016, Eriksson, 2016). Research has found that stronger SOC is associated with improved health outcomes, including lower stress and better tension management (Amirkhan and Greaves, 2003), healthier behavior and lifestyle choices (Wainwright et al., 2007), and reduced risk of all-cause mortality (Piiroinen et al., 2020, Super et al., 2014). Strengthening SOC may therefore be a valuable way of improving a variety of health outcomes. However, it is unclear if SOC can be strengthened in adulthood. Although SOC was initially hypothesized to be stable after age thirty (e.g. Antonovsky, 1987), there is growing evidence that health-focused interventions may strengthen SOC among adults (e.g. Ley and Rato Barrio, 2013, Schreuder et al., 2014). This may be particularly the case if SOC is low prior to the intervention (Vastamäki et al., 2009). We were therefore interested in assessing the extent to which SOC strengthened during an intervention aimed at older, vulnerable adults.

How might SOC be strengthened? According to the salutogenic perspective of health, this occurs via Generalized Resistance Resources (GRRs). GRRs are social and individual resources that ‘help to manage stress and to thrive, moving towards the positive end of an ease/dis-ease continuum’ (Antonovsky, 1979). GRRs can be divided into material, genetic, knowledge-based, and social domains (Super et al., 2016). Repeated use of GRRs may strengthen SOC, and vice versa. Interventions aimed at strengthening GRRs may therefore strengthen SOC.

One pathway to strengthen GRRs, and consequently SOC, is experiential learning, as argued by Schreuder et al. (2014). Experiential learning is defined as learning in which knowledge is created through the ‘grasping and transforming of experience’ (Kolb, 1984). Experiential learning occurs when four processes are present: experiencing, reflecting, thinking, and acting. These processes are thought to empower individuals and to help them move toward a more efficacious coping style (Super et al., 2016). Interventions incorporating aspects of experiential learning may therefore help to strengthen GRRs and SOC.

However, it is not yet clear what type of interventions are most effective at engaging in experiential learning, and ultimately at strengthening SOC. To date, interventions have ranged from those focused on nutrition (Forsberg et al., 2010); care farming (Schreuder et al., 2014), and mindfulness (Humboldt and Leal, 2013, Kähönen et al., 2012). Only one study has examined change in SOC during a physical activity intervention, and found a significant strengthening in SOC after the intervention (Ley and Rato Barrio, 2013). This represents a knowledge gap, given that physical activity is an important way to engage in experiential learning. However, there is less direct evidence that physical activity interventions may strengthen SOC. Sharma et al. (2006) argued that physical activity fosters improvements in mental health because the activity itself fosters distraction, self-efficacy, and social interaction. There is also evidence that increased physical activity plays a role in improved tension management, which may help to strengthen SOC (Huang et al., 2013). Further, Hassmén et al. (2000) found that individuals who exercised more had stronger SOC scores. Moreover, this relationship may be reciprocal: experiencing stress has been shown to decrease physical activity (Stults-Kolehmainen and Sinha, 2014).

In this study, we therefore investigated whether SOC may have strengthened in the context of a Dutch community-based physical activity intervention aimed at vulnerable older adults. We hypothesized that this was indeed the case, particularly among individuals whose SOC at baseline was weak.

## 2 Methods

### 2.1 Setting

This study focuses on the intervention, Communities on the Move. In the intervention, participants were, via purposive sampling, recruited in collaboration with the Knowledge Center for Sport & Physical Activity Netherlands, and with representatives from local programs. These local program representatives were approached through the Knowledge Center for Sport & Physical Activity Netherlands network, information meetings, training sessions, field visits and snowball procedures. Participation was on a voluntary basis. Most participants in the intervention were from low SES backgrounds, and/or were immigrants to the Netherlands (Herens, 2016). Moreover, Communities on the Move targeted older adults (age 50+). Ethics approval for Communities on the Move was obtained from the Social Sciences Ethics Committee at Wageningen University and Research.

Experiential learning was embedded in Communities on the Move. Participants gave input into recruitment, program design, and tailoring physical activities to their needs. Participants practiced what they learned, and actively involved their social and physical environments, in order to sustain their behavior change (Herens et al., 2015). This means the actual content of the groups and programs varied.

The data used in this study came from the evaluation study of Communities on the Move (Herens et al., 2013, Herens, 2016). Participants were recruited and monitored in four sequential cohorts. Data collection for cohort 1 started in autumn 2012, and for cohort 4 in spring 2014. Information on SOC was collected alongside a number of indicators of effectiveness, including physical activity behavior, health related quality of life, self-efficacy, social support and physical activity enjoyment (Herens et al., 2013). Table 1 presents an overview of the number of participants, groups and programs.

**Table 1:** Overview of ommunities on the Move programs.

**Program**	**Municipality**	**Target group**	**Program design**	**Gender**	**# groups**	**# participants**
1	Amsterdam	• socially vulnerable • non-Dutch origin	• fixed duration (10 weeks) • outdoor • walking/running • 1x/week • multiple exercise trainers	women	1	14
2	Den Haag	• socially vulnerable • non-Dutch origin	• continuing • in-/outdoor • exercise to music/fall prevention/walking • 1x/week • one known exercise trainer	women	3	31
3	Enschede	• socially vulnerable • Dutch and non-Dutch origin	• fixed duration (13 weeks + 18 months follow-up meeting every 6 weeks) • in-/outdoor • mixed sport activities • 1x/week • multiple exercise trainers	women men	2 1	30
4	Helmond	• socially vulnerable • Dutch and non-Dutch origin	• continuing • outdoor • outdoor fitness • multiple times/week • one known exercise trainer	mixed	2	39
5	Hengelo	• socially vulnerable elderly (55+) • Dutch and non-Dutch origin	• fixed duration (12 weeks) • in-/outdoor • mixed sport activities • 1x/week • multiple exercise trainers	women men	3 1	51
6	Rotterdam	• socially vulnerable elderly • mostly non-Dutch, some Dutch origin	• continuing • indoor • exercise to music/fall prevention • multiple times/week • one known exercise trainer	women men	3 1	73
7	Tilburg	• socially vulnerable or chronically ill elderly (55+) • Dutch origin	• continuing • indoor • fall prevention exercises/mixed sport activities • 1x/week • one known exercise trainer	women mixed	1 1	30

*Source:*Herens (2016).

The structure and duration of the programs varied. While some lasted for a fixed duration (10–13 weeks), other programs took the form of ongoing physical education classes. These exercises included outdoor activities (e.g. walking, running, outdoor fitness) and indoor activities (e.g. endurance training, dance, Zumba) (Herens et al., 2015). At baseline, 268 participants were included, who were active in 19 groups (of 10–20 participants) distributed over seven Dutch municipalities (Herens et al., 2013). For all cohorts, data were collected in four waves: T_0_, T_1_ at six months, T_2_ at twelve months, and T_3_ at 18 months. At T_3_, there were 117 participants with complete covariate information. Data were collected via pen and paper questionnaires and were in Dutch, the working language of Communities on the Move. Socio-demographic factors and measurements of health, including SOC, were measured at baseline. SOC was measured again only at T_3_.

### 2.2 Variables

#### 2.2.1 Sense of coherence

Our key predictor was SOC at baseline (T_0_). We derived SOC scores from the SOC-3 questionnaire, comprised of three questions, with one each aimed at manageability, comprehensibility, and meaningfulness (Lundberg and Peck, 1995). These questions (from the original SOC-3 questionnaire, and translated to Dutch) were asked as follows: “Do you usually see a solution to problems and difficulties that others see as hopeless?” (manageability); “Do you usually find that the things that happen to you in everyday life are difficult to understand?” (comprehensibility); “Do you usually find that your daily life is a source of personal satisfaction?” (meaningfulness) (*ibid.*). Each question was scored by the participant from 1 to 3, whereby a score of 1 was associated with a strong SOC, and a score of 3 with a weak SOC. The combined SOC score therefore had a minimum of 3 (very strong SOC) and a maximum of 9 (very weak SOC). However, given this study’s small sample size, the condensed, three-category version of SOC-3 at T_0_ was used as the key predictor in regressions. Here, a score of 3 was considered strong, a score of 4 or 5 was considered moderate, and a score of 6 through 9 was considered weak (*ibid.*).

**Fig. 1:**
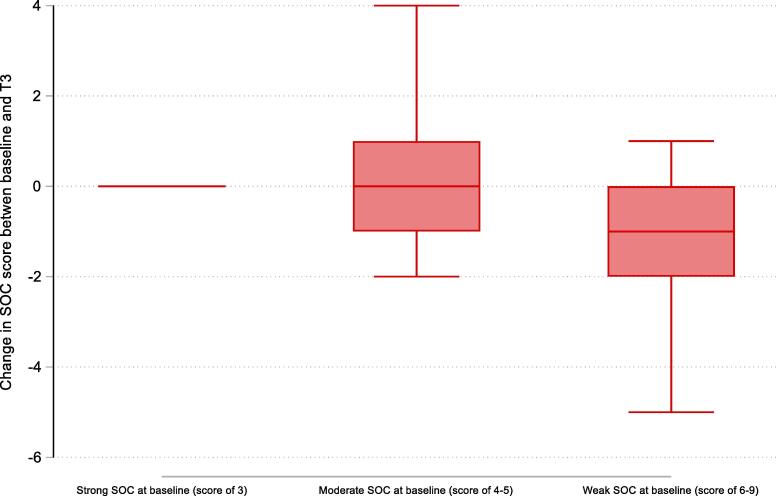
The change in SOC between baseline and T_3_, by SOC score category at baseline.

The outcome of this study was change in SOC score. This was measured by differencing SOC at the final wave of the study (T_3_) and at baseline (T_0_). This more extended scale was used in Fig. 1, to understand the extent to which SOC changed. However, due to data sufficiency considerations, a three-category variable was used as the outcome in the main regression analyses, whereby: 1 = strengthening (Δ SOC score < 0), 2 = remaining the same (Δ SOC score = 0), and 3 = weakening (Δ SOC score > 0) between T_0_ and T_3_.

#### 2.2.2 Covariates

Demographic characteristics were included as covariates. All covariate information was self-reported and taken from baseline measurements. These covariates were: education, age, BMI, having an immigrant background, gender, and smoking.

### 2.3 Analyses

#### 2.3.1 Main analyses

Data were analyzed in Stata version 16. We first calculated sample characteristics for all variables included in our analyses. This included a descriptive graph of the relationship between SOC score at T_0_ and SOC score at T_3_. Then, using a pretest–posttest design (e.g. Clifton and Clifton, 2019), we performed several regressions to better-specify the change in SOC: unadjusted and adjusted ordinal logistic regressions; and an adjusted ordinal mixed regression with random intercepts for location and groups, in order to assess whether location and group were associated with change in SOC score. Results were reported as odds ratios, whereby an odds ratio greater than one represented an increased odds of having weaker SOC at T_3_. For the ordinal logistic regressions, pseudo-R^2^s were reported. For the mixed model, the variance component parameters of groups and locations were reported. Also reported was the result of a likelihood-ratio test, a measure of goodness-of-fit, between the adjusted mixed ordinal logistic regression and the adjusted ordinal logistic regression. Finally, the marginal estimates of the probability of SOC changing between T_0_ and T_3_ were calculated, and were presented graphically.

#### 2.3.2 Robustness check

We did not have information on SOC at T_3_ (or any other point after baseline) for those who dropped out of Communities on the Move. Out of the initial 268 participants, only 117 finished the program. It may be that those who dropped out were systematically different from those who finished the intervention. To assess whether this was the case, logistic regressions were performed, in order to test if SOC at baseline was associated with an increased odds of dropping out.

## 3 Results

### 3.1 Sample characteristics

Sample characteristics are presented in Table 2. At baseline (T_0_), 16% of participants had strong SOC scores (scores of 3). Fifty-six percent of participants had moderate SOC scores (scores of 4 or 5) at T_0_. Further, 27% of participants had weak SOC scores (scores of 6 through 9). At T_3_, the largest share of participants (65%) reported no change in SOC. This is followed by 21% reporting stronger SOC scores. An additional 14% reported weaker SOC scores.

**Table 2:** Sample characteristics.

	**Obs.**	**%**
***Difference in SOC score between T_0_ and T_3_*:**		
Stronger SOC (negative change)	25	21.37
No change in SOC	76	64.96
Weaker SOC (positive change)	16	13.68

***SOC score at baseline (T_0_):***		
Strong SOC (Score of 3)	19	16.24
Moderate SOC (Score of 4–5)	66	56.41
Weak SOC (Score of 6–9)	32	27.35

***Education:***		
Primary/no education	46	39.32
Secondary education and above	71	60.68

***Income assistance:***		
No response	29	24.79
Receiving income assistance	43	36.75
Not receiving income assistance	45	38.46

***Age:***		
<50 years	22	18.42
50–64 years	40	34.21
65–74 years	34	29.82
>75 years	21	17.54

***BMI:***		
Normal weight	28	23.93
Overweight	41	25.04
Obese	48	41.03

***Born in the Netherlands:***		
Yes	58	50.43
No	59	49.57

***Gender:***		
Man	15	12.82
Woman	102	87.18

***Smoking status:***		
Non-smoker	14	11.97
Previous smoker	33	28.21
Smoker	55	47.01
Unknown	15	12.82

***Location of program:***		
Amsterdam (Group 5)	2	1.71
Den Haag (Groups 15, 16, 17)	15	12.82
Enschede & Hengelo (Groups 8, 10, 13, 14, 18, 19)	22	18.80
Helmond (Groups 2, 7)	19	16.24
Rotterdam (Groups 3, 4, 6, 9)	38	32.48
Tilburg (Groups 1, 11)	21	17.95

Fig. 1 depicts the median change in SOC score, based on SOC at baseline. Those with weak SOC scores at T_0_ (with scores between 6 and 9) experienced the largest strengthening of SOC: these individuals’ SOC scores strengthened (decreased) by a median score of one point. In comparison, participants with strong SOC scores (with scores of 3) or moderate SOC scores (with scores of 4 or 5) at baseline reported a median change of zero points between T_0_ and T_3_.

### 3.2 Main results

**Fig. 2:**
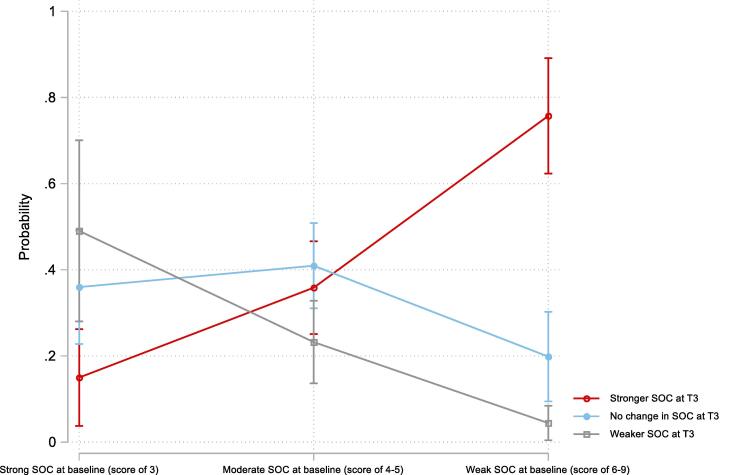
Marginal estimates of the change in SOC score, by SOC at baseline.

Table 3 presents the main results. Across all models, having a strong SOC score at baseline (scores of 3) was strongly, significantly associated with an increased odds of SOC score weakening at T_3_, relative to the reference group of having a moderate SOC score (scores of 4 or 5) at T_0_. Conversely, having a weak SOC score at baseline (scores of 6 through 9) was strongly, significantly associated with a decreased odds of SOC weakening at T_3_. Fig. 2 presents the marginal estimates of the adjusted ordinal mixed regressions. Having a weak SOC at baseline was associated with a 76% probability of stronger SOC at T_3_, and a 4% probability of weaker SOC at T_3_. There was therefore evidence in support of this study’s hypothesis that SOC score strengthened during Communities on the Move.

**Table 3:** SOC at baseline’s relationship to SOC at T3.

	Unadjusted ordinal logistic regression results				Adjusted ordinal logistic regression results				Adjusted ordinal mixed regression results			
	OR	p-value	95% confidence interval		OR	p-value	95% confidence interval		OR	p-value	95% confidence interval	
***SOC score at baseline (T_0_):***												
Strong SOC (Score of 3)	2.106	0.101	0.864	5.135	3.962	0.023	1.212	12.950	3.962	0.023	1.212	12.951
Moderate SOC (Score of 4–5)	Ref	Ref	Ref	Ref	Ref	Ref	Ref	Ref	Ref	Ref	Ref	Ref
Weak SOC (Score of 6–9)	0.206	0.001	0.083	0.511	0.127	0.000	0.043	0.370	0.127	0.000	0.043	0.371

***Education:***												
Primary/no education					1.066	0.900	0.392	2.898	1.066	0.900	0.392	2.897
Secondary education and above					Ref	Ref	Ref	Ref	Ref	Ref	Ref	Ref

***Income assistance:***												
No response					3.355	0.030	1.123	10.020	3.355	0.030	1.123	10.020
Receiving income assistance					Ref	Ref	Ref	Ref	Ref	Ref	Ref	Ref
Not receiving income assistance					0.782	0.651	0.269	2.271	0.782	0.651	0.269	2.271

***Age:***												
<50 years					1.221	0.745	0.367	4.067	1.221	0.745	0.367	4.067
50–64 years					Ref	Ref	Ref	Ref	Ref	Ref	Ref	Ref
65–74 years					1.423	0.490	0.522	3.884	1.423	0.490	0.522	3.884
>75 years					0.828	0.756	0.252	2.718	0.828	0.756	0.252	2.718

***BMI:***												
Normal weight					3.124	0.032	1.105	8.833	3.124	0.032	1.105	8.834
Overweight					Ref	Ref	Ref	Ref	Ref	Ref	Ref	Ref
Obese					0.723	0.517	0.270	1.932	0.723	0.517	0.270	1.932

***Born in the Netherlands****:*												
Yes					0.192	0.010	0.055	0.672	0.192	0.010	0.055	0.672
No					Ref	Ref	Ref	Ref	Ref	Ref	Ref	Ref

***Gender:***												
Women					0.546	0.300	0.174	1.713	0.546	0.300	0.174	1.713
Men					Ref	Ref	Ref	Ref	Ref	Ref	Ref	Ref

***Smoking status:***												
Non-smoker					Ref	Ref	Ref	Ref	Ref	Ref	Ref	Ref
Previous smoker					1.312	0.598	0.478	3.604	1.312	0.598	0.478	3.604
Smoker					0.817	0.752	0.234	2.858	0.817	0.752	0.234	2.858
Unknown					2.620	0.175	0.652	10.527	2.620	0.175	0.652	10.530
												
***Group*** (variance component)									0.000			
***Location*** (variance component)									0.000			
***Pseudo R***^***2***^	0.104				0.162				n/a			
***LR test (mixed ordinal vs ordinal logistic regression)***	n/a				n/a				0.12	0.367

Regarding the results of the mixed ordinal regression, the variance component parameters of group and program location were both 0.000. Also, based on the results of a likelihood-ratio test, the mixed model was not a better fit than the ordinal logistic regression. Therefore, group and program location do not appear to have played a role in the change in SOC score.

### 3.3 Robustness check results

After adjusting for covariates, SOC score at baseline was not significantly related to the odds of dropping out of Communities on the Move. However, receiving income assistance, and giving no response to this question were significantly associated with an increased odds of dropping out of Communities on the Move. Being older and being born in the Netherlands were associated with lower odds of dropping out of Communities on the Move. These results are available on request.

## 4 Discussion

In this study, we explored the potential for SOC to be strengthened during the intervention, Communities on the Move. Our study stood out for several reasons. First, we examined change in SOC during a physical activity intervention, a relatively understudied area. Second, our sample stood out for its participants: they were, on the one hand, from vulnerable backgrounds, and therefore potentially had the greatest likelihood of improving SOC; on the other hand, study participants were older, and therefore were thought to have relatively stable SOC scores. We found evidence that SOC strengthened between baseline and T_3_, and that program group and location did not explain the variance in changes in SOC. These findings chimed with existing research, which has argued that there is more potential to change SOC among vulnerable groups, because these groups have the most to gain (e.g. Hochwälder, 2019, Vastamäki et al., 2009). Overall, our findings reinforced that SOC is reflective of a major life orientation that is difficult but possible to change, particularly among those with initially weak SOC (Lindstrom and Eriksson, 2005).

Our results support the argument made by Schreuder et al., (2014), that interventions that explicitly incorporate experiential learning may help to strengthen SOC. As noted, experiential learning was embedded in Communities on the Move at the individual, group and program location levels. There is evidence that experiential learning can occur at both individual and group levels, with these different levels reinforcing one another (Fragkos, 2016). The processes of experiencing, reflecting, thinking and acting may have resulted in stronger GRRs and SOC scores.

The importance of experiential learning overall, versus group and location specifics, may be why accounting for clustering at the group and location level did not better explain the change in SOC between T_0_ and T_3_, relative to not accounting for clustering. As noted, there was substantial variation in the content and duration of the Communities on the Move intervention groups (Herens et al., 2015). The overarching experience of participating in Communities on the Move appeared to have played a more important role in strengthening SOC, than group or location specifics.

This study also provided evidence that SOC may be a useful indicator for health promotion interventions. Traditionally, health promotion interventions have solely utilized objective measures of success, e.g. weight loss in physical activity interventions. However, these measures may only capture short-term improvements in behavior change, versus long-term changes in health. Withall et al. (2014) argued that subjective measures of health may indicate potential further improvements in health, and are therefore important to collect. Given that SOC has been found to be associated with health-promoting behaviors (e.g. Wainwright et al., 2007), an improvement in SOC may mean that participants are more likely to experience improved health in the long-term.

Moreover, using subjective indicators like SOC may be particularly important when interventions do not show improvements in physical activity (Marcus et al., 2000). A relatively common issue for physical activity interventions is that they do not demonstrate a change in physical activity behavior, or participants report a return to baseline activity levels after interventions have concluded (Craike et al., 2018, Dunn et al., 1998, van Woerkum and Bouwman, 2014). Similarly, Communities on the Move participants on average did not report significant changes in physical activity levels (Herens, 2016). Yet, it appears that participants did benefit from taking part in Communities on the Move, with improved SOC scores as an indication of this. Using SOC as an indicator with which to evaluate physical activity interventions may help to paint a more complete picture of interventions’ successes.

Further, this study provided evidence that using an abbreviated SOC scale may be an appropriate and valid way to measure SOC. In Communities on the Move, SOC was measured via the three-item SOC-3 scale for practical reasons. There are well-established logistical challenges to using more elaborated SOC scales, particularly regarding the relatively long time it takes to complete them, and interview respondents’ difficulty in understanding the questions (Lundberg and Peck, 1995). Incomplete answers result in missing items, with the resulting sum score being excluded from analysis (Naaldenberg et al., 2011). The SOC-3 scale was developed to address these problems. However, compared to other SOC scales, the SOC-3 scale may be less sensitive to changes in SOC (Schumann et al., 2003). Still, Togari et al. (2007) argued that the SOC-3 scale showed some convergent and concurrent validities with more elaborate SOC scales. Further, if indeed the criticisms of the SOC-3 scale’s validity are accurate, then we should expect a larger change in SOC with a more elaborate scale (Piiroinen et al., 2020). Given that we found a sizeable, significant change in SOC, this does not appear to have been a problem in our study.

### 4.1 Limitations

However, this study was not without limitations. Data were derived from a multiple case, multiple level cohort study to measure effectiveness and processes simultaneously (Herens et al., 2013). As a consequence, a limitation of this study is the absence of a control group, due to the absence of appropriate ways to define comparable control groups in real life settings. Further, non-observable differences, such as initial motivation, are difficult to match in practice (Herens, 2016, Koelen et al., 2001). However, it is worth bearing in mind that this study could not definitively establish whether SOC changed due to participation in Communities on the Move, because of the absence of a control group.

This study also had a relatively small sample size, with 117 participants completing Communities on the Move. It may be that this study’s findings are particular to the study sample, and therefore should be interpreted with some caution. However, in terms of the validity of these results, the small sample size should not be cause for concern: studies with smaller sample sizes are more prone to type II error than type I error (Columb and Atkinson, 2016). In a larger sample, we accordingly should expect even greater strengthening of SOC scores than what was found in this study.

Similarly, this study had a high rate of drop-outs: 43% of participants at T_0_ were present at T_3._ However, once we accounted for baseline characteristics, we did not find significant differences in SOC at baseline between those who dropped out and those who completed the program. Still, there is some cause for concern: Herens et al. (2016) compared other indicators collected during Communities on the Move, including physical activity levels, health-related quality of life, self-efficacy and enjoyment outcomes, measured at 12 months in the program, between drop-outs and non-drop outs. This previous study found that, when comparing other indicators of well-being, those who dropped out tended to score less positively. Moreover, in the present study, receiving income assistance (or not reporting a response) and being born abroad were significantly associated with the likelihood of dropping out. Ultimately, we found some evidence that more vulnerable participants were more likely to drop out.

This pattern of drop-outs is by no means unique to Communities on the Move: in general, more vulnerable individuals are both less likely to be recruited for health promotion interventions, and are less likely to complete interventions once they are involved (Linke et al., 2011, Smit et al., 2021). Communities on the Move explicitly targeted recruitment to more vulnerable individuals, and did not appear to suffer from these recruitment problems. However, perhaps clearly-defined strategies to keep individuals involved, and more active follow-up among drop-outs could have helped to minimize the differences among those who completed Communities on the Move and those who did not. This has already been found to be effective with recruitment: in a systematic review, Cooke and Jones (2017) found that studies with active tactics (i.e. targeting lower-income individuals) were more successful than those with passive tactics. Applying such tactics in practice, of course, requires time and money, and is easier said than done (Smit et al., 2020). However, these active strategies may help to lessen, rather than reproduce and magnify, existing socio-economic iniquities in health promotion.

## 5 Conclusion

In this study, we offered further insights into how and why SOC may be strengthened during a physical activity intervention. We found that SOC strengthened over the course of Communities on the Move, with those with the weakest SOC scores at baseline experiencing the largest strengthening in SOC. This study therefore provided evidence that SOC may be possible to strengthen among adults, particularly among those whose SOC scores are initially low. Based on the fact that change in SOC did not vary across groups and programs with different physical activity content, we argued that the intervention itself – rather than program-specific factors – played a larger role in strengthening SOC. Ultimately, SOC, as a subjective measures of well-being, may be an important complementary indicator to health promotion interventions.

## Funding

The completed evaluation study of Communities on the Move was funded by the ZonMW project, “Effectiveness and cost-effectiveness of the Communities on the Move program” (project number: 200130010).

## CRediT authorship contribution statement

**Kristina Thompson:** Methodology, Formal analysis, Writing – original draft, Writing – review & editing, Visualization. **Marion Herens:** Conceptualization, Project administration, Data collection, Writing – original draft, Writing – review & editing. **Johan van Ophem:** Conceptualization, Writing – review & editing. **Annemarie Wagemakers:** Conceptualization, Project administration, Supervision, Writing – review & editing.

## Declaration of Competing Interest

The authors declare that they have no known competing financial interests or personal relationships that could have appeared to influence the work reported in this paper.
